# The Role of High Mobility Group Box 1 Protein (HMGB1) in the Immunopathology of Experimental Pulmonary Tuberculosis

**DOI:** 10.1371/journal.pone.0133200

**Published:** 2015-07-22

**Authors:** Rogelio Hernández-Pando, Jorge Barrios-Payán, Dulce Mata-Espinosa, Brenda Marquina-Castillo, Diego Hernández-Ramírez, Oscar Adelmo Botasso, Estela Isabel Bini

**Affiliations:** 1 Experimental Pathology Section, Department of Pathology, National Institute of Medical Sciences and Nutrition “Salvador Zubirán”, México City, 14000, México; 2 Immunology Department, National Institute of Medical Sciences and Nutrition “Salvador Zubirán”, México City, 14000, México; 3 Institute of Experimental and Clinic Immunology, Rosario, IDICER, CONICET, School of Medical Sciences, Santa Fe 3100, Rosario, 2000, Argentina; Fundação Oswaldo Cruz, BRAZIL

## Abstract

**Background:**

The high mobility group box 1 (HMGB1) is the prototype of alarmin protein released by stressed or dying cells. The redox state of this protein confers different functions in the regulation of inflammation and immune response.

**Aim:**

Determine the kinetics, cellular sources and function of HMGB1 in experimental tuberculosis.

**Methods:**

BALB/c mice were infected with *Mycobacterium tuberculosis* strain H37Rv. At different time points, HMGB1 was quantified in bronchial lavage fluid (BALF) and in lungs was determined its cellular sources by immunohistochemistry. HMGB1 was blocked with specific antibodies or recombinant HMGB1 was administered during early or late infection. Bacilli burdens, inflammation and cytokines expression were determined.

**Results:**

The maximal concentration of HMGB1 in BALF was at day one of infection. Bronchial epithelium and macrophages were the most important sources. At day 7 to 21 the oxidized HMGB1 was predominant, while during late infection only the reduced form was seen. Blocking HMGB1 during early infection produced significant decrease of bacilli burdens and high production of pro-inflammatory cytokines, while the opposite was seen when HMGB1 was administered. Blocking HMGB1 activity or administrated it in high amounts during late infection worsening the disease.

**Conclusions:**

HMGB1 is liberated during experimental tuberculosis and promotes or suppress the immune response and inflammation depending on the redox state.

## Introduction

Tuberculosis (TB) is a respiratory chronic infection which produces profound abnormalities in the immune system [1]. Both innate and acquired immunity are essential participants in the growth control of *Mycobacterium tuberculosis* (Mtb). During early infection, innate immunity senses the presence of the pathogen after the participation of a number of pattern-recognition receptors that detect mycobacterial components though pathogen-associated molecular patterns (PAMPs), being the Toll-like receptors (TLRs) the best studied of these pattern detectors. Interestingly, besides to recognizing PAMPs, the immune system has evolved to detect endogenous danger signals or by analogy damage-associated molecular patterns (DAMPs), which are released by dying cells or are actively secreted by stressed cells and contributes to regulate the inflammatory response [2]. Actually DAMPs act as ‘warning’ signals that alert innate and adaptive immunity. The nuclear DNA-binding molecule high mobility group box 1 (HMGB1) is a prototype DAMP protein that may play a role in modulating the inflammatory responses after the cell damage induced by Mtb [3].

HMGB1 is a non-histone nuclear protein that is comprised of 215 amino acids that are arranged in two box structures (A box and B box) and a C terminal tail with glutamic and aspartic aminoacids. HMGB1 contains three cysteine residues, two in box A, (C23 and C45), and one in box B (C106) that are redox sensitive, and two nuclear localization sequence (NSL) located one in the box A and the other one in box B, both contain lysine residues. Hyperacetylation of the lysines located in NSLs determines the nuclear translocation to cytoplasm and subsequent secretion [4]. Thus, acetylation is decisive for intracellular shuttling of HMGB1 from the nucleus to cytoplasm and subsequent release from monocytes, macrophages [5, 6] and other cell types [4].

In the nucleus, HMGB1 can bind DNA, especially molecules with certain sequences or a bent structure, contributing to organize chromosome architecture and regulates transcription [7, 8]. In the cytoplasm, HMGB1 is involved in autophagy and PKR/inflammosome activation [4]. HMGB1 is susceptible to extensive post-traslationals modifications: acetylations, methylations, glycations, phosphorylations, ADP rybosilations, and reversible and terminal cysteine oxidation [4, 9, 10, 11]. HMGB1 can enter endosomal vesicles for eventual secretion after immune activation or other type of stimulus.When cells die by necrosis or apoptosis, HMGB1 also translocates to the extracellular milieu [3, 12], and its immunological effect is different.When HMGB1 is liberated by necrotic cells induces strong pro-inflammatory stimulus, as demonstrated in models of sepsis [13], while HMGB1 released during apoptosis could diminish immunological activity, due to the oxidation of key cysteine residues occurring during redox disturbances in stressed cells [14].

Recent analysis based in mass spectrometry, molecular techniques and immunological readouts have allowed the functional characterization of HMGB1, which depends on the redox modifications of cysteine residues and lysine acetylation [4].Concerning to the cysteine residues and depending on the redox state, HMGB1 can be in “all thiol” form with all cysteines reduced; “disulfide HMGB1” with a disulfide bond between C23 and C45, and C106 remaining in the reduced thiol form; and the “oxidized HMGB1” with the three cysteines oxidized [15, 16, 17]. The “all thiol” HMGB1 acts as a chemotactic mediator [4], after binding to other chemokines (CXCL-12), it stimulates leukocyte recruitment [15, 18]. The “disulfide” HMGB1 is a cytokine-stimulating factor, it is released by necrotic and pyroptotic cells, and binds to MD-2 in the TLR4/MD-2 complex inducing TNFα release and NFκβ activation acting as a proinflammatory factor [4, 17], while oxidized HMGB1 is released by apoptotic cells and induces immunosuppressing /antinflammatory effects [15, 16, 17, 4].

Considering that along the course of TB there are necrotic, apoptotic and stressed cells which should release HMGB in different redox states, the contribution of this alarmin in the immunopathology of TB could be important.The present study is aimed to evaluate the kinetics, cellular sources and function of HMGB1 in a model of pulmonary TB in BALB/c mice.

## Materials and Methods

### Experimental model of pulmonary TB

The experimental model of progressive pulmonary TB has been described elsewhere [19]. Briefly, the reference Mtb strain H37Rv was growth in 7H9 medium with OADC enrichment. Mid log-phase cultures were used. Male BALB/c mice, 6–8 weeks old, were anaesthetized in a gas chamber using sevofluorane and infected through endotracheal instillation with 2·5 x 10^5^ live bacilli. All the animal work was done according to the guidelines of the Mexican Constitution law NOM 062-200-1999, and approval of the Ethical Committee for Experimentation in Animals of the National Institute of Medical Sciences and Nutrition in Mexico, permit number: 224.

### Quantification of HMGB1 by ELISA and determination of reduced or oxidized HMGB1 by western blotting in bronchial lavage fluid (BALF)

Groups of four animal infected as described below were euthanized by ex-sanguination after anesthetized with intraperitoneal penthotal; BALF was performed with 5×1 ml of PBS on 1, 3, 7, 14, 21, 28 and 60 days after infection. Another group of uninfected animals was included as a control group. After concentrate the BALF, total proteins from mice were determined separately and HMGB1 concentrations were determined by ELISA using a commercial kit (IBL International, REF: ST 51011).

The presence of reduced or oxidized HMGB1 was determined in BALF, because depending on this is its pro or anti-inflammatory activity [2, 20]. HMGB1 oxidation produced a shift in the protein emigration which can be detected in gradient polyacrylamide gels and western-blotting [20]. Equal amounts of BALF proteins from each time point were separated on a non-reducing (4 to 20%) Bis-Tris gel (Invitrogen, Carlsbad, CA), transferred to nitrocellulose membrane and incubated with anti-HMGB1 Rabbit anti-Human Monoclonal Antibody (LS- B11655-LSBio) diluted 1/1000. The blot was developed by chemiluminescence (ECL-plus; Amersham-Pharmacia, Piscataway, NJ). Control blots were processed without incubation with the primary antibodies.

### Preparation of lung tissue for immunohistochemistry and morphometry

Lungs from infected and non infected mice were fixed by perfusion with 100% ethanol via the trachea and embedded in paraffin. Sections 5μ thick were deparaffinized, the endogenous peroxidase quenched and incubated with anti-HMGB1 Rabbit anti-Human Monoclonal Antibody diluted 1/250 in PBS (LS-B11655-LSBio), followed by incubation with goat anti-rabbit IgG labeled with peroxidase. The same antibodies used in the western-blot study were used in the immunohistochemistry experiment. Bound antibodies were detected with diamino-bencidine. For quantification, three different mice lungs per time-point in two different experiments were evaluated. Ten random microscopy fields were selected. At x200 magnification, at least 1200 negative or positive cells from the airways epithelial cells and alveolar and interstitial macrophages were counted using an image analyser (Leica Q-win, Cambridge UK) [19]. Due to the results obtained with the administration of recombinant HMGB1 during early and late infection, immunohistochemistry to detect foxP3 and IL-10 were also performed using polyclonal specific antibodies (Santa Cruz Biotechnology), following the same described procedure.

The subcellular localization of HMGB1 was determined by immunoelectronmicroscopy using antibodies labeled with colloidal gold. Lungs from three mice from days 1, 7 and 60 days after infection were perfused with 4% (v/v) paraformaldehyde dissolved in 0·2 M Sörensen buffer (1 vol NaH2PO4*H2O, 2 vol NaHPO4*7H2O) for 4 h at 4°C. Tissue fragments were dehydrated and embedded in LR-white hydrosoluble resin (London Resin Company, London, UK). Sections from 70 to 90 nm on nickel grids were incubated overnight with monoclonal rabbit anti-HMGB1 antibodies diluted 1/20. Then, the grids were incubated with goat anti-rabbit antibodies conjugated with 5 nm gold particles (Sigma Co., St Louis, MO), contrasted with uranium salts (Electron Microscopy Sciences, Fort Washington, PA) and examined with the electron microscope. As negative controls, the primary antibody was substituted by normal rabbit serum.

### Real-time PCR analysis of cytokines in lung homogenates

One lung lobe, from three different mice per group in two independent experiments, was used to isolate mRNA using the RNAeasy minikit (Qiagen). Quality and quantity of RNA were evaluated through spectrophotometry (260/280) on agarose gels. Reverse transcription of the mRNA was performed using 5 μg RNA, oligo (dT), and the Omniscript kit (Qiagen, Inc.). Real-time PCR was carried out using the 7500 real time PCR system (Applied Biosystems) and Quantitect SYBR green Mastermix kit (Qiagen). Standard curves of quantified and diluted PCR product, as well as negative controls, were included in each PCR run. Specific primers for genes encoding acidic ribosomal protein (RLP0) as house-keeping gene, tumor necrosis factor alpha (TNFα), gamma interferon (IFNγ), interleukin 17 (IL-17) and interleukin 10 (IL-10) were designed using the program Primer Express (Applied Biosystems) [21]. Cycling conditions were: initial denaturation at 95°C for 15 min, followed by 40 cycles at 95°C for 20 s, 60°C for 20 s, and 72°C for 34 s. Data are shown as copies of cytokine-specific mRNA/10^6^ copies of RLP0 mRNA.

### Effect of blocking HMGB1 with specific antibodies or administration of recombinant HMGB during early TB infection

In order to study the contribution of HMGB1 to the inflammatory and immune response during early TB, infected mice were treated with chicken blocking antibodies against HMGB1(Chicken Anti-HMGB1 Polyclonal Antibody, Cat N° 326052233 Shino Teste Corporation, Japan), administrating 15 μg/100 μl PBS by intratracheal route, on days 1, 3, 6, 9 and 12 after infection and groups of three mice in two independent experiments were euthanized at days 1, 7, 14 and 28 post-infection. Control mice received the same amount of isotype chicken antibodies (IgY, Shino TesteC, Kanawaga Japan). The lungs were processed for bacilli loads determination by colony forming units counting; the measurement of inflammatory infiltrates in square microns in different lung compartments (around venules, bronchioles and alveolar-capillary interstitium), granuloma size and percentage of lung surface affected by pneumonia were determined using automated morphometry (Q Win, Leica, Milton Keynes, UK). Pneumonia corresponded to lung areas with widening of alveolar capillary interstitium due to abundant inflammatory infiltrate and intra-alveolar proteinaceous material intermixed with inflammatory exudates, where numerous macrophages are located. In order to quantifie pneumonia, the total lung area was measured, then all the areas affected by pneumonia were determined and the percentage of affected lung surface area was calculated. Cytokines (TNFα, IFNγ, IL-17 and IL-10) gene expression was determined by RT-PCR as previously described [21].

In other group of mice the bacilli burdens and the inflammatory and immunological responses were evaluated after intratracheal administration of recombinant HMGB1 (Human, His-tagged, recombinant, Baculovirus. ATGEN Cat N° HMG0801). Since day one of infection and each other day during the first month of infection, mice received by intratracheal route 30ng/ml of recombinant HMGB suspended in PBS. Control group received the same amount of human albumin in the same indicated days. Groups of three mice in two independent experiments were euthanized on days 1, 3, 7, 14, 21 and 28 days post-infection and their lungs were processed as described above. The amount of administered HMGB1 was the same than the maximal detected by ELISA, trying to maintain a constant high concentration of this protein during early infection.

### Effect of blocking HMGB1 with specific antibodies or administration of recombinant HMGB1 during late disease

Groups of mice after 60 days of infection were treated with blocking antibodies against HMGB1, 15 μg/100μl administered intratracheally each other day during three weeks and mice were euthanized one month later. Other group of mice was treated with 30ng of recombinant HMGB1 suspended in 50μl of PBS, administered each other day by intratracheal route during two months. Control group received the same amount of IgY or human albumin instead of blocking antibodies or recombinant HMGB-1 respectively. Animals were euthanized after two months of treatment and their lungs were studied as described above.

### Statistical Analysis

Data are presented as the mean +/-standard deviation. Differences among groups were evaluated by the Anova test, whereas the Student t test was used for further analysis among group differences. An associated probability lower than 0.05, was considered significant.

## Results

### Kinetics of HMGB1 in BALF during the course of progressive pulmonary TB

**Fig 1:**
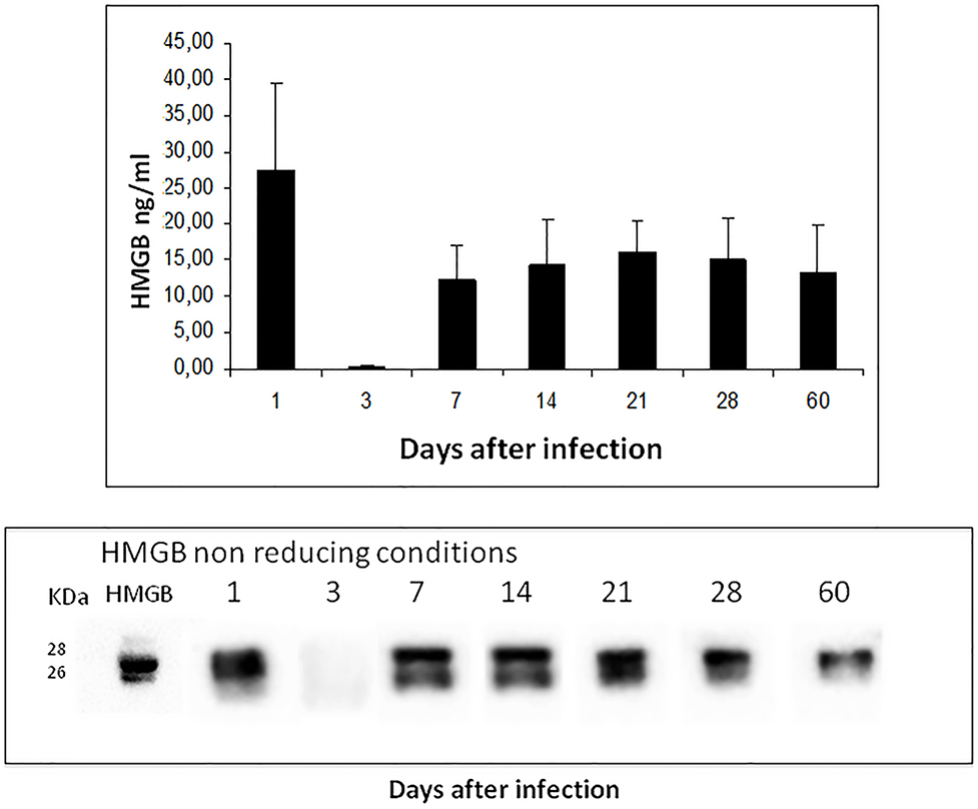
Kinetics of HMGB1 production during the course of experimental pulmonary tuberculosis. BALB/c mice were infected with a high dose of *M*. *tuberculosis* strain H37Rv, groups of four animal per indicated time point were euthanized and their lungs were processed for the determination of HMGB1 in bronchioalveolar lavage proteins by ELISA (top panel), and identification of the reduced (one band) or oxidased (double bands) forms of HMGB1 (bottom panel). As control reduced recombinant human HMGB is shown.

The quantification of HMGB1 in BALF by ELISA showed the highest concentration on day one of infection, followed by a sharp decrease on day three. After one week and until two months of infection there was a constant concentration of HMGB1 in BALF, from 10 to 15ng/ml (Fig 1). HMGB1 detection by Western-blot in BALF showed a strong band on day one post-infection, which almost disappear on day 3; from day 7 to 21 the double band corresponding to the oxidized form of HMGB1 was clearly detected, while during progressive disease only the single band corresponding to the reduced form was observed (Fig 1).HMGB-1 was not detected in BALF from non-infected animals by ELISA or Western-blot studies (data not shown).

### Cellular sources and subcellular localization of HMGB1 determined by immunohistochemistry

**Fig 2:**
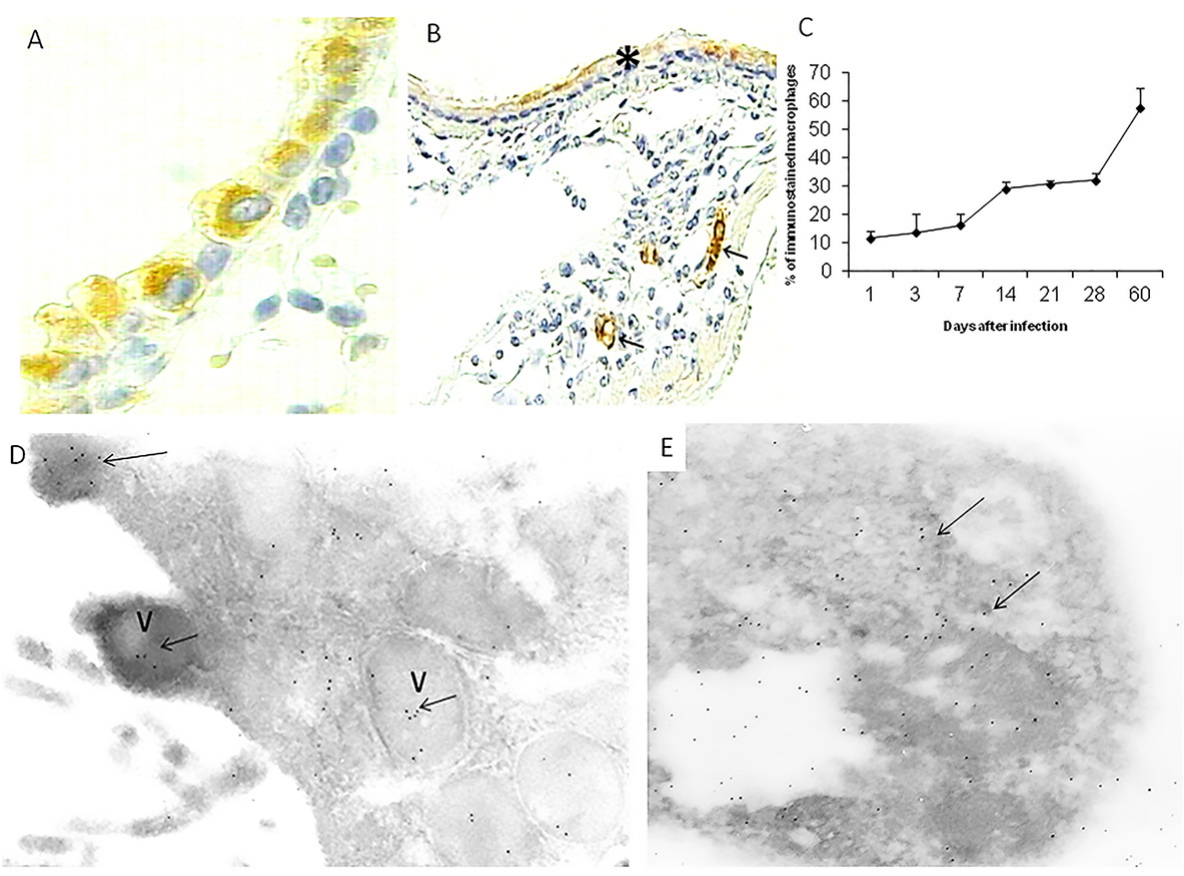
Cellular sources of HMGB1 determined by immunohistochemistry. (A) Lung section from a mouse after one day of infection, there is strong HMGB1 immunostaining in the cytoplasm of bronchial epithelial cells. (B) After 60 days of infection, the bronchial epithelium show scarce immunereactivity (asterisk), while macrophages in the pneumonic areas show strong immunostaining (arrows). (C) The morphometric study shows a progressive increase of macrophages with HMGB1 immunostaining. (D) Immunoelectronmicroscopy micrograph from a bronchial epithelial cell after one day of infection, there are several submembranal vesicles (V) with HMGB1 detected with antibodies labeled with colloidal gold (arrows). (E) An apoptotic macrophage from day seven of infection shows many black dots that correspond to HMGB1 detected with specific antibodies labelled with colloidal gold (arrows).

Lung sections from non-infected mice showed occasional bronchial epithelial cells with scare cytoplasmic immunostaining. In contrast, during the early phase of the infection the most common HMGB1 immunostained cells were the bronchial epithelium with a constant 70–80% of positive cells determined by automated morphometry (Fig 2A), while at late disease occasional epithelial cells showed scarce immunoreactivity (Fig 2B). Alveolar and interstitial macrophages also showed HMGB1 immunostaining during early infection and progressively increased, being 10 +/- 3% on day one to 30+/-5% on day 60 of infection in the pneumonic areas (Fig 2C); occasional positive cells were found in granulomas. HMGB1 immunostained macrophages showed the morphology of activated cells: very large cells with abundant eosinophilic cytoplasm and big nucleus with prominent nucleoli.

The immunoelectronmicroscopy study showed in the bronchial epithelial cells some nuclear labeling and cytoplasmic vesicles with specific labeling; some of these vacuoles were connected to the apical membrane (Fig 2D). On days 1 and 7 of infection, numerous apoptotic macrophages were seen with strong labeling (Fig 2E).

### Effect of blocking HMGB1 or recombinant HMGB1 administration during early infection

**Fig 3:**
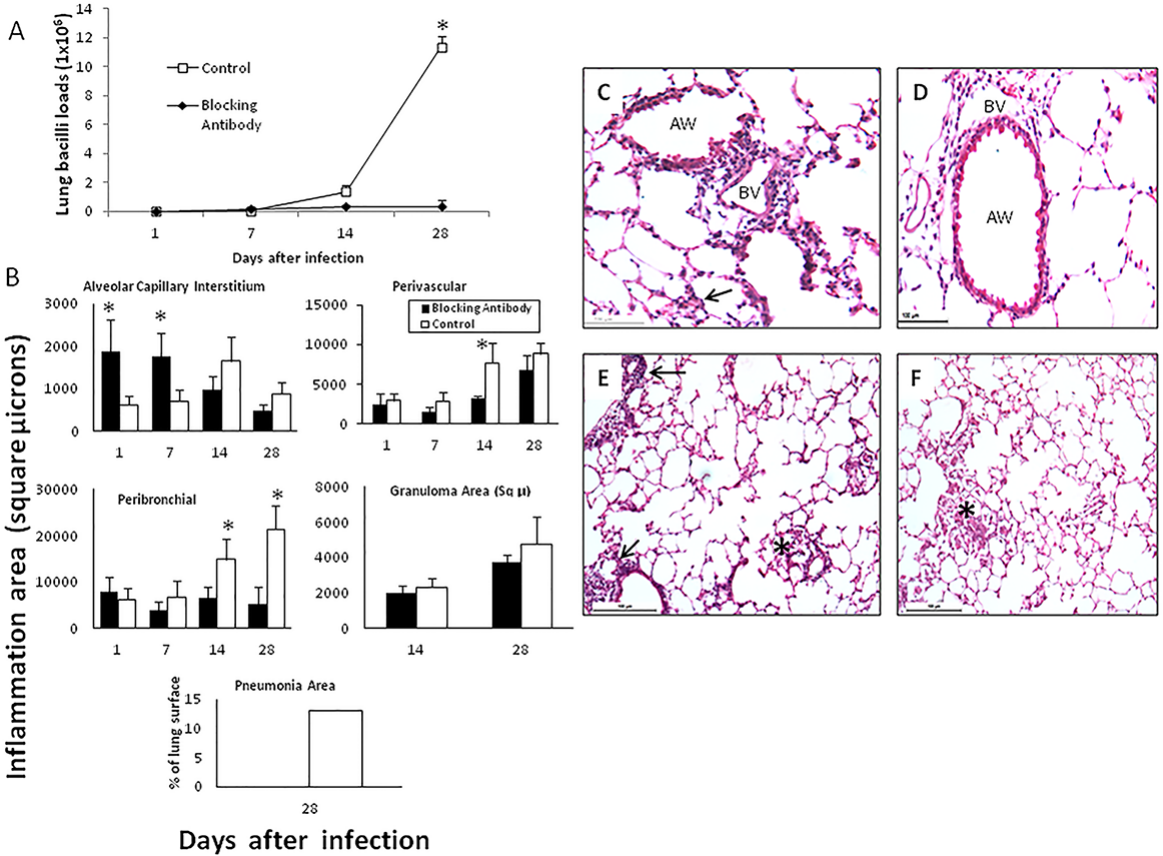
Effect of blocking HMGB1 during early infection on bacilli loads and inflammation. (A) Groups of infected BALB/c mice with *M*.*tuberculosis* strain H37Rv were treated with blocking specific antibodies against HMGB1 administered each other day since the first day of infection and during three weeks (solid symbols); control animals received isotype irrelevant chicken antibodies (white symbols). (B) The lungs from three mice per each sacrifice day were used to determine the area in square microns occupied by inflammatory cells in the indicated lung compartment, in mice treated with HMGB1 blocking antibodies (black bars) and control group (white bars). Asterisks represent statistical significance (p<0.05 student T test). (C) Representative lung micrograph from a mouse treated with blocking HMGB1 antibodies after one week of infection, there are numerous lymphocytes around blood vessels (BV), airways (AW) and alveolar-capillary interstitium (arrow). (D) In contrast, control mouse show lower inflammation in the same lung compartments after 7 days of infection. (E) After one month of infection, the lung of mouse treated with blocking antibodies shows high inflammatory response around blood vessels (arrows) and in the interstitium (asterisk) but there is not pneumonia. (F) In comparison, the lung of control mouse show focal patches of pneumonia (asterisk). (All figures H/E staining, scale bar = 100μ).

In order to study the contribution of HMGB1 to immunopathology during early *M*. *tuberculosis* infection (first month), infected mice were treated with HMGB1 blocking antibodies during the first three weeks of infection. These animals showed similar lung bacilli loads than control mice treated with irrelevant isotype chicken antibodies after one week of infection. Then, significant lower bacilli burdens were seen in animals treated with blocking antibodies (Fig 3A). In comparison with control mice, animals that received HMGB1 blocking antibodies showed higher inflammation in the alveolar-capillary interstitium during the first week of infection. Then, on days 14 and 28, higher inflammation in all lung compartments was developed by control mice, probably as a consequence of the higher bacilli burdens (Fig 3B). The size of granulomas was similar in both treated and non-treated mice. Focal areas of pneumonia that affected 10+/-3% of the total lung surface area was observed in control animals on day 28 of infection, while treated mice did not show pneumonia, probably as a consequence of the high bacterial elimination produced by the HMGB1 blocking antibodies administration (Fig 3C–3F).

**Fig 4:**
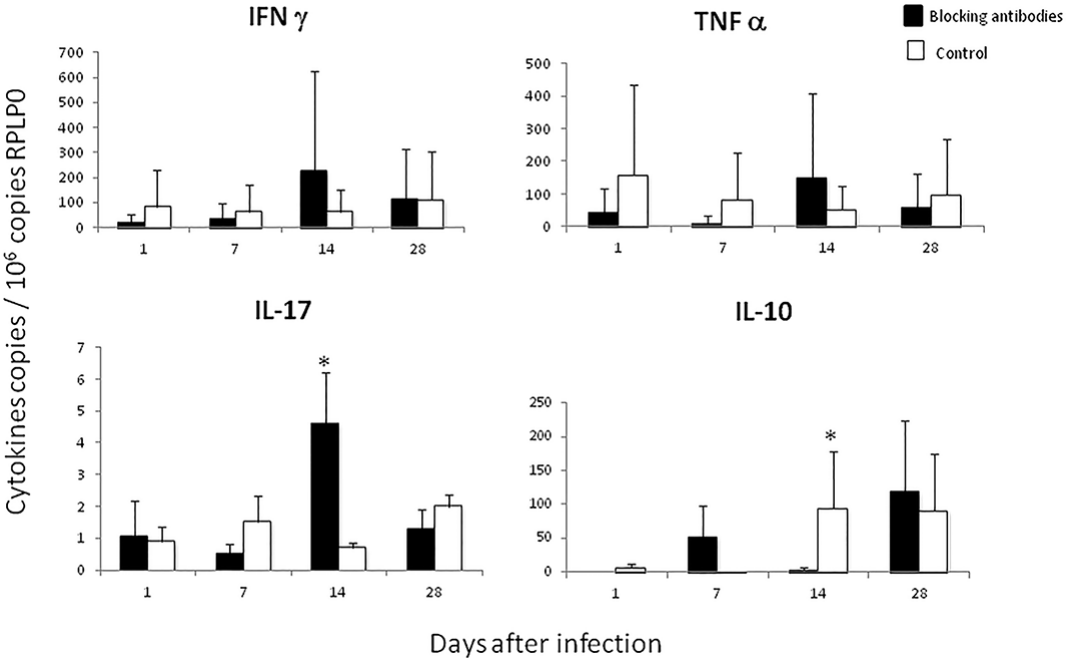
Effect of blocking HMGB1 during early infection on cytokines expression. Quantitative expression of mRNA for the indicated cytokines was determined by real-time PCR in lungs from mice treated with HMGB1 blocking antibodies (black bars) or control animals (white bars). Data are expressed as means and standard deviations for four different animals at each time point. Asterisks represent statistical significance (*P* 0.05 Student T test).

The determination of cytokines expression showed in animals treated with HMGB1 blocking antibodies lower expression of the pro-inflammatory cytokines IFNγ, TNFα and IL-17 during the first week of infection, while at day 14 the expression of these proinflammatory cytokines increased and the transcription of IL-10 was significant lower than control mice. At day 28 similar cytokines expression was seen in both groups (Fig 4).

**Fig 5:**
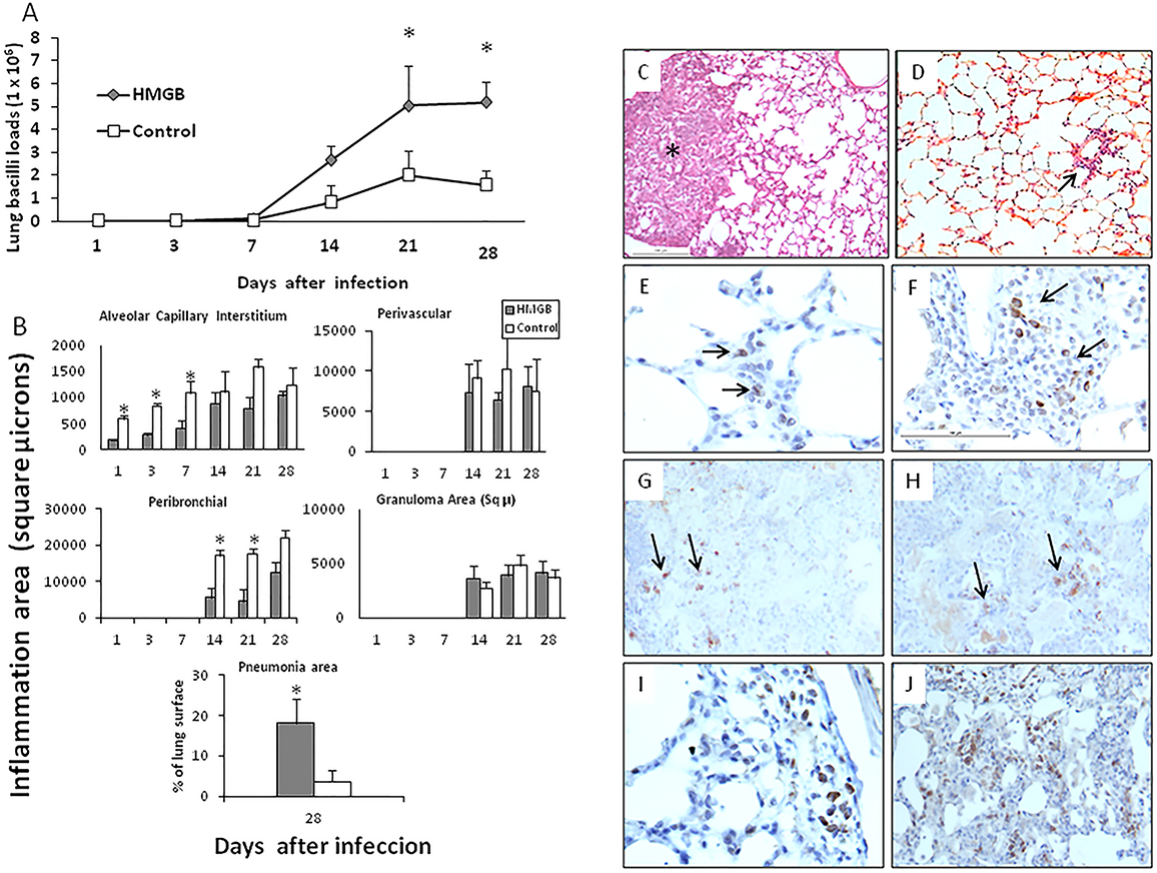
Effect of administrate recombinant HMGB1 during early infection on bacilli burdens and inflammation. (A) Groups of infected BALB/c mice with *M*.*tuberculosis* strain H37Rv were treated with recombinant human HMGB1 administered each other day by intratracheal route since the first day of infection and during three weeks (solid symbols); control animals received human albumin (white symbols). (B) The lungs from three mice per each sacrifice day were used to determine the area in square microns occupied by inflammatory cells in the indicated lung compartment in mice that received by intratracheal route recombinant human HMGB1(black bars) or human albumin (control group,white bars). Asterisks represent statistical significance (p<0.05 student T test). Representative micrographs of lungs from mice treated with recombinant HMGB1 or control animals: (C) After 28 days of infection, treated mouse with recombinant HMGB1 show areas of pneumonia (asterisk). (D) In contrast, control mouse at the same time of infection show perivascular inflammation (arrow), without pneumonic areas. (E) Ocassional cells show foxP3 immunostaining around blood vessels (arrows) in control mice at 7 days of infection. (F) In comparison, more inflammation and foxP3 immunostained cells (arrows) are seen in mice treated with recombinant HMGB1 at 14 days of infection. (G) Control mouse show some foxP3 immunostained cells in the pneumonic areas after 60 days of infection (arrows). (H) The same areas show some IL-10 immunostained cells (arrows). (I) The lung of a mouse treated with recombinant HMGB1 during late infection show more foxP3 immunostained cells around blood vessel and in the pneumonia areas (J).

**Fig 6:**
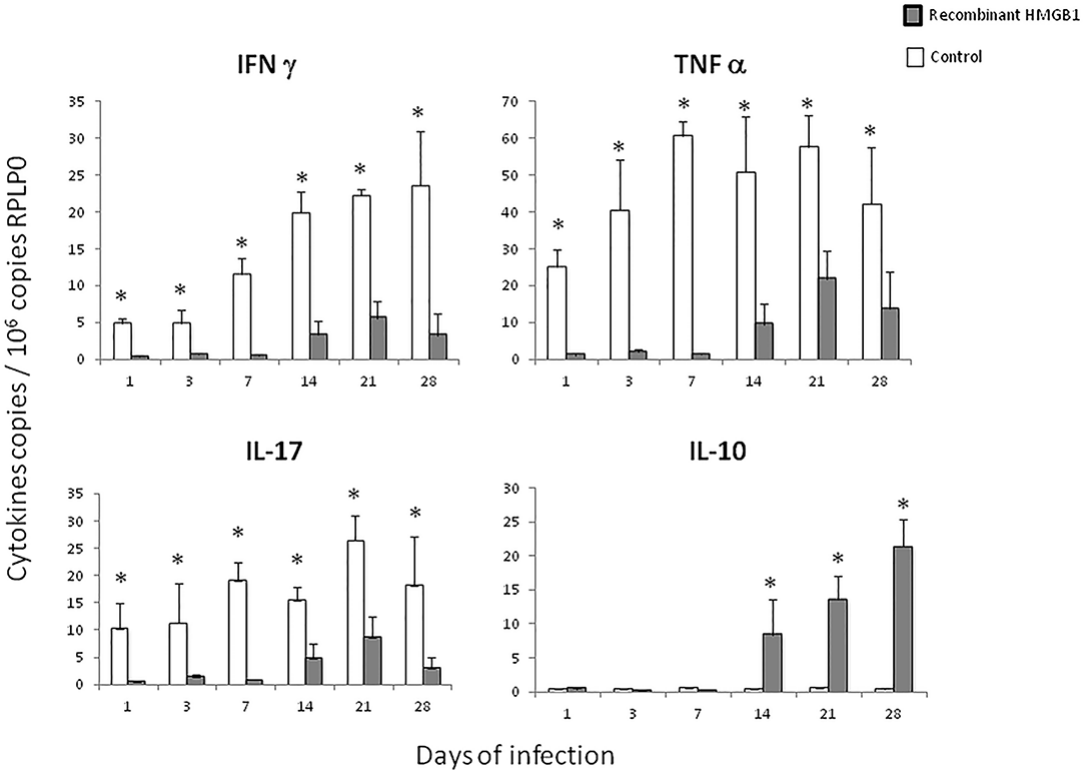
Effect of the intratracheal administration of recombinant HMGB1 during early infection on cytokines expression. Quantitative expression of mRNA for the indicated cytokines was determined by real-time PCR in lungs from mice that received recombinant human HMGB1 (black bars) or control animals (white bars). Data are expressed as means and standard deviations for four different animals at each time point. Asterisks represent statistical significance (P 0.05 Student T test).

In comparison with control animals that received human albumin, mice that received HMGB1 by the intratracheal route showed a significant increase of bacilli burdens after one week of infection (Fig 5A). These treated animals also showed a decrease of inflammation in all lung compartments, being significant in the alveolar-capillary interstitium during the first week of infection (Fig 5B). Similar granuloma size was measured in both groups, but animals that received HMGB1 developed significant more pneumonia on day 28 of infection (Fig 5B and 5C). In comparison with the control group, mice treated with recombinant HMGB1 showed significant lower expression of IFNγ, TNFα and IL-17 in all time points and significant higher IL-10 expression at 14, 21 and 28 days of infection (Fig 6), with numerous immunostainedcells with antibodies against foxP3 and IL-10 (Fig 5I and 5J).

### Effect of blocking HMGB1 or recombinant HMGB1 administration during late disease

**Fig 7:**
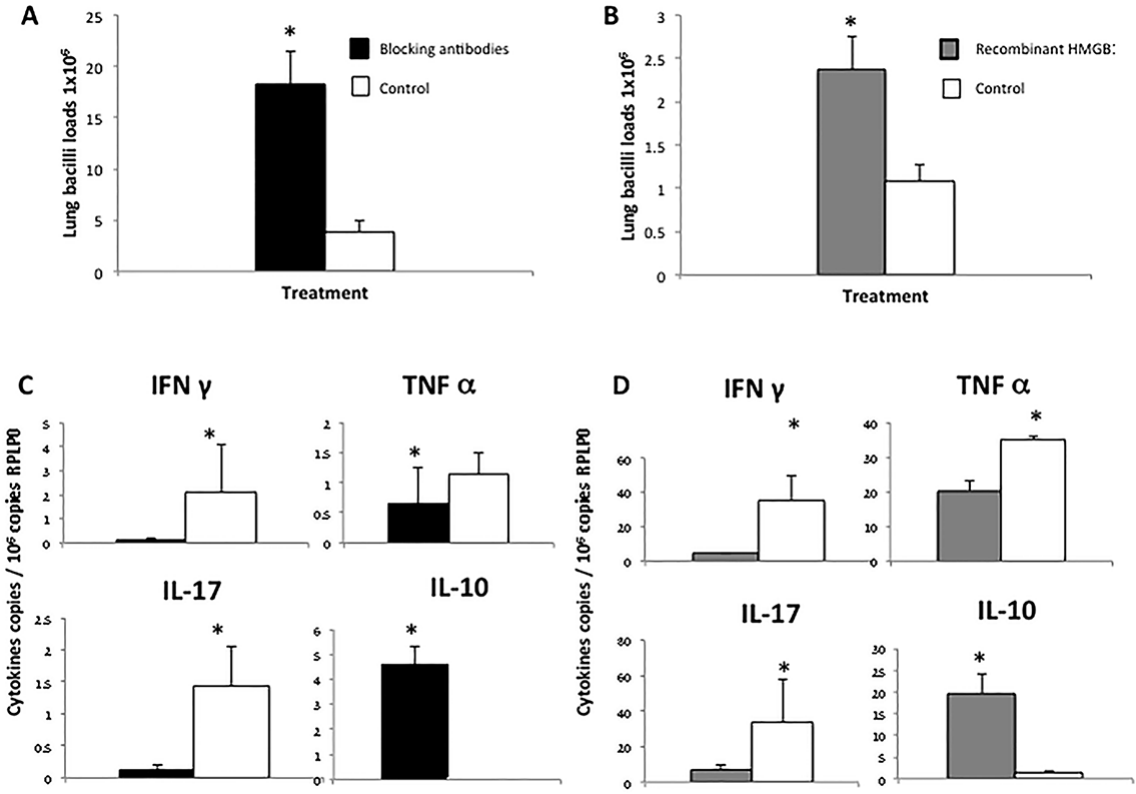
Effect of the administration of blocking antibodies against HMGB1 or recombinant human HMGB1 during late disease on pulmonary bacilli loads and cytokines expression. (A) Bacilli burdens in lungs of mice after 60 days of infection treated during one month with HMGB1 blocking antibodies (black bars) or isotype chiken antibodies as a control (white bars). (B) Pulmonary bacilli loads of BALB/c mice after 60 days of infection with *M*. *tuberculosis* strain H37Rv treated during one month with recombinant human HMGB1 (grey bars) or human albumin as a control (white bars) (6 mice per group). (C) Quantitative expression of mRNA of cytokines determined by real-time PCR in lungs from mice after 60 days of infection and treated with HMGB1 blocking antibodies administered each other day during one month and control non-treated animals. (D) Cytokines transcription during late disease in mice treated with recombinant human HMGB1 or non-treated control animals. Asterisks represent statistical significance between control (white bars) and treated groups (grey bars) (p<0.05 student T test).

During late active disease, numerous macrophages showed HMGB1 immunostaining and mild concentrations in BALF of this protein in its reduced proinflammatory form were seen (Figs 1 and 2). In order to study the contribution of HMGB1 to the evolution of TB during late progressive phase, specific blocking antibodies were administered after two months of infection and during one month. In comparison with the control group, animals that received blocking HMGB1 antibodies after 60 days of infection showed a significant increase of pulmonary bacilli burdens (Fig 7A), lower expression of IFNγ, TNFα and IL17, high expression of IL-10 (Fig 7C), and higher but not significant pneumonia (39+/3% vs 32+/3%).

In order to study the course of the infection during late TB with similar high concentrations of HMGB than those found during early infection, a high concentration of recombinant HMGB1 was administered after 60 days of infection by intratracheal route during one month. In comparison with control mice, animals that received recombinant HMGB1 showed fivefold higher bacilli loads (Fig 7B), the double of pneumonia and significant lower expression of IFNγ, TNFα and IL-17, while the expression of IL-10 was significantly higher (Fig 7D). Treated mice also showed numerous foxP3 and IL-10 immunostained cells than control animals (Fig 5I and 5J).

## Discussion

HMGB1 is highly conserved with >99% amino acid identity between murine and human molecules. HMGB1 is extensively modified posttranslationally [8, 22, 23]. Acetylation and phosphorylation induce HMGB1 translocation from the nucleus to the cytoplasm and increase its secretion by inflammatory cells [5]. HMGB1 is passively released from necrotic cells and is actively secreted by stressed and inflammatory cells, binding with high affinity to several receptors, such as RAGE, TLR-2, TLR-4, TLR-9, and CD24 as a negative signaling molecule [24, 25]. In a previous study, was demonstrated that the infection *in-vitro* of macrophages and monocytes with mycobacteria induced HMGB1 release, and some mycobacterial proteins such as 65kD HSP induced its maximum release. This study also reportedHMGB1 released in lungs during infection with Mtb in guinea pigs, and concluded that HMGB1 may acts as a signal of cellular injury that enhances immune response [26]. Our study confirms and extends these results by the demonstration that during progressive pulmonary TB in BALB/c mice, there is an active and high secretion of HMGB1 during early infection, followed by a mild and constant production during late disease, but our results suggest that this protein has different activity regulating the immune and inflammatory response depending of the infection phase.

Active secretion of HMGB1 has been demonstrated in many cell types, such as monocytes, macrophages, dendritic cells (DC), hepatocytes, endothelial cells, glial cells and neurons [3]. Our ELISA assay in BALF did not show HMGB-1 in non-infected mice, while a rapid and high secretion of HMGB1 raising its highest level after the first day of infection was seen followed by a sharply decrease on day three and increased again on day seven; but it was three fold lesser than day one and this mild concentration was maintained constantly until late disease. Thus, it seems that preformed HMGB1 is rapidly liberated particularly from the airways epithelium which showed the strongest HMGB1 immunostaining during early infection; also the immunoelectronmicroscopy study indicated active secretion, as suggested by the observation that some cytoplasmic vesicles with labeled HMGB1 were fused to the apical membrane liberating the protein to the extracellular space. Numerous activated and apoptotic macrophages showed HMGB1 immunostaining during early infection. Thus, elements of the innate immunity such as bronchial epithelium and macrophages are the most important source of HMGB1 during early TB infection, and airways epithelium should be added to the list of cells that can actively secrete this protein.

Particularly when HMGB1 is liberated from necrotic cells and after binding to RAGE and Toll-4 receptors, it induces maturation of dendritic cells (DC) and secretion of IL-12 and IFNγ that polarizing toward a Th1 phenotype [27]. Interaction with TLR4 is required for HMGB1 activation of cytokine release from macrophages. Stimulation of neutrophils and monocytes with HMGB1 induces cytokine release and promotes migration into inflamed tissue [28], as well as endothelium activation [29]. All these activities could be related to an efficient Mtb elimination. Interestingly, HMGB1 is also released by apoptotic cells inducing immune tolerance [14, 30, 31]. During apoptosis, HMGB1 is oxidized on Cys106 in Box B, a process that requires caspase activity and mitochondrial reactive oxygen species (ROS) [14]. This oxidized HMGB1 is unable to interact with RAGE and Toll-4 receptors, avoiding its pro-inflammatory activity [32]. Oxidized HMGB1 can also induce immune-modulation by the recruitment and activation of T regulatory cells [33].

The oxidation of HMGB1 in the cysteine residue 106 produced a shift in the protein emigration, which can be detected in gradient polyacrylamide gels and western-blotting [20]. We detected oxidized HMGB1 in BALF on days 7, 14 and 21 postinfection, when in this model there are numerous activated and apoptotic macrophages [34].Thus, it seems that during early Mtb infection, there is an oxidative environment in the lung that favoring the production of the oxidized HMGB1 with immune-toleragenic activity. This was confirmed by the administration of specific blocking antibodies since the first day of infection. Blocking HMGB1 activity during the first week of infection induced lower expression of TNFα, IFNγ and IL-17, denoting certain HMGB1 pro-inflammatory activity, but on day 14, when the oxidized form of HMGB1 was produced and its activity was blocked, a significant decrease in pulmonary bacilli loads was seen in coexistence with higher expression of pro-inflammatory cytokines. As expected, the opposite response was observed when recombinant human HMGB1 was administrated during early infection, inducing a significant increase of bacilli loads, in coexistence with low expression of proinflammatory cytokines and a striking high expression of IL-10 and numerous Treg cells in the infected lungs, which confirm the efficiency of HMGB1 in the recruitment and activation of this cell type [35]. We observed numerous IL-10 immunostained macrophages, suggesting that high concentrations of HMGB1 could also stimulate the production of this anti-inflammatory cytokine by macrophages.

During late disease, on day 28 and 60 of infection, there are extensive areas of pneumonia, the bronchial epithelium in these zones showed an accentuated decrease of HMGB1 immunostaining and macrophages were the most common and intense immunoreactive cells. During advanced disease, there was mild concentration of HMGB1 in BALF and the oxidized form was not detected. The administration of blocking antibodies at day 60 of infection induced a significant increase of pulmonary bacilli burdens, in coexistence with lower expression of pro-inflammatory cytokines. These results suggest that the mild production of reduced HMGB1 during late disease has proinflammatory activity and contributes in the control of bacilli growth. Interestingly, the administration of recombinant HMGB1 in high concentrations, similar than found at day one of infection, induced significant higher bacilli loads, more extensive tissue damage, lower expression of proinflammatory cytokines with high expression of IL-10, in coexistence with numerous T regulatory cells, suggesting that not only the type of HMGB1, reduced or oxidized, but also the amount of the protein is important to regulate the immune response and its interaction with specific subtypes of T cells. Indeed, T regulatory cells express on their cell membrane more RAGE than T conventional cells, and HMGB1 induces the migration and prolonging survival of T-regulatory cells, as well as enhance IL-10 release [35]. In addition, HMGB1 can directly suppress IFNγ release by effector T cells and inhibits its proliferation via TLR-4 in the setting of chronic inflammatory states [33, 35, 36], such as produced by Mtb infection.

In conclusion, HMGB1 is liberated during experimental TB and can modify the fate of the immune response, promoting or suppressing inflammation depending on redox state and its concentration. During early infection there is a highly oxidizing environment produced by numerous activated macrophages that actively produce ROS and NO [37], and apoptotic macrophages that liberate oxidized HMGB1, that temporally suppress excessive inflammation and decrease protective immunity. During late disease, the oxidative environment decreases as a consequence of lesser macrophage activation with lower ROS and NO production as well as lesser macrophage apoptosis [34, 37], HMGB1 is produced in lesser amount and it is not oxidized contributing to the control of bacilli growth. Thus, HMGB1 is a redox-sensitive protein that is affected by the oxidative environment which modulates its pathophysiological signals and contribution to the control of inflammation and immune response against mycobacterial infection.
