# Correction: The Role of High Mobility Group Box 1 Protein (HMGB1) in the Immunopathology of Experimental Pulmonary Tuberculosis

**DOI:** 10.1371/journal.pone.0137263

**Published:** 2015-08-27

**Authors:** Rogelio Hernández-Pando, Jorge Barrios-Payán, Dulce Mata-Espinosa, Brenda Marquina-Castillo, Diego Hernández-Ramírez, Oscar Adelmo Bottasso, Estela Isabel Bini

The sixth author’s name is spelled incorrectly. The correct name is: Oscar Adelmo Bottasso. The correct citation is: Hernández-Pando R, Barrios-Payán J, Mata-Espinosa D, Marquina-Castillo B, Hernández-Ramírez D, Bottasso OA, et al. (2015) The Role of High Mobility Group Box 1 Protein (HMGB1) in the Immunopathology of Experimental Pulmonary Tuberculosis. PLoS ONE 10(7): e0133200. doi:10.1371/journal.pone.0133200

The following information is missing from the Funding section: This work was also supported by the Immunology of Cancer and Infectseases network, CONACYT N° 253053.
